# Generation of mixed ovarian cancer and fibroblast spheroids by bioprinting

**DOI:** 10.1007/s44164-026-00107-z

**Published:** 2026-03-03

**Authors:** Arjan Atwal, Alan Richardson

**Affiliations:** Guy Hilton Research Centre, School of Allied Health Professions and Pharmacy, Keele University, Staffordshire, ST4 7QB UK

**Keywords:** Cancer-associated fibroblasts (CAFs), Bioprinting, 3D tumour model, Collagen hydrogel, Tumour–stroma, Luciferase, Carboplatin, Ovarian cancer

## Abstract

Three-dimensional tumour–stroma models are urgently needed to improve the predictive power of preclinical drug screening in ovarian cancer, a disease with high mortality and limited therapeutic success. Here, we present a droplet-bioprinted ovarian cancer model incorporating OVCAR-4 cells expressing nano-luciferase and primary cancer-associated fibroblasts (CAFs) embedded within a collagen (Telecol)-based hydrogel. Using the CELLINK BIO X system, we generated reproducible mini-spheroids with controlled architecture, enabling systematic evaluation of cancer and CAF cell density and chemotherapeutic response. Bioprinted spheroids showed good reproducibility across plates when bioink volume and printing conditions were stable. Increasing cancer cell density produced a non-linear rise in luminescence, and this could be at least partially corrected by normalisation to initial cell number. CAFs consistently and robustly enhanced cancer cell growth in a ratio-dependent manner, and confocal imaging revealed that CAFs frequently organised into encapsulating structures around cancer clusters, forming stromal niches associated with matrix contraction and increased cancer–CAF interface. However, CAFs did not protect cancer cells against carboplatin, as drug sensitivity remained comparable in monoculture and CAF co-cultures. This suggests that CAF-driven growth support and CAF-driven chemoresistance are mechanistically separable in this model. Together, this work establishes a mechanically tunable, spatially defined bioprinted ovarian cancer–CAF model that captures key stromal interactions which are not reproduced in studies using CAF conditioned media alone, while remaining compatible with automated, higher-throughput workflows.

## Introduction

Cancer remains a major global health challenge, accounting for millions of deaths each year despite decades of advances in early detection and therapy. Among all malignancies, ovarian cancer stands out for its particularly poor prognosis. It is often diagnosed at an advanced stage, when the disease has already disseminated within the peritoneal cavity. The high rates of relapse following chemotherapy and the development of drug resistance leads to limited treatment options and often prevents long-term cures. Whilst the development of new therapeutics offers hope, 90% of all drugs that enter clinical trials, not just those used to treat ovarian cancer, fail to reach the market. This suggests that current pre-clinical models are inadequately predictive of clinical outcomes. This, and the recent trend to reducing animal testing, notably led by the FDA, highlights a need for the development of improved in vitro models [1, 2].

To address these limitations, researchers have developed three-dimensional (3D) spheroid models which better recapitulate gradients of oxygen, nutrients, and metabolites. While spheroids more closely mimic key aspects of in vivo tumours, their spontaneous formation via self-aggregation often results in significant variability in size, cellular composition, and reproducibility. These shortcomings hinder the scalability required for systematic drug screening. Flörkemeier et al. recently demonstrated that co-culturing ovarian cancer cells with fibroblasts in ultra-low-attachment plates produces more compact and physiologically relevant spheroids [3]. However, this approach relies on spontaneous self-assembly without an extracellular matrix and lacks scalability, as spheroids must be formed manually in ultra-low-attachment plates. As a result, it cannot adequately capture ECM–cell interactions, collagen remodelling, produce defined construct geometries and it may not be suited to high-throughput drug testing.

Recent advances in bioprinting technologies offer an innovative solution by enabling the controlled deposition of cancer cells and extracellular matrix components into uniform 3D constructs. Bioprinting can generate large numbers of reproducible mini-tumors with defined architecture, facilitating high-throughput experimentation under physiologically relevant conditions. Incorporating co-culture systems into these models further enhances their relevance [4]. In ovarian cancer, cancer-associated fibroblasts (CAFs) play a pivotal role in disease progression, supporting cancer cell survival, promoting dedifferentiation to stem-like states, and mediating drug resistance [5]. By providing metabolic and paracrine support, CAFs can significantly influence treatment outcomes. Therefore, recreating these stromal interactions in vitro is essential to faithfully model tumour biology. Some studies have assessed the effect of CAFs simply by adding CAF conditioned media to cultures of cancer cells, but this totally fails to capture the effect of direct cell-cell interactions. Thus, true co-cultures of CAFs and ovarian cancer cells are required. However, a method is needed to specifically measure the effect of drugs on the cancer cells in spheroids comprising a mixed population of cell types; traditional metabolic readouts would be inappropriate because they would measure the entire cell population.

Herein, we describe the development of a bioprinted co-culture model of ovarian cancer comprising OVCAR-4 cancer cells, stably expressing nano-luciferase and a red fluorescent protein for tracking cancer cell growth, and CAFs embedded within a collagen-based hydrogel. The constructs were generated using the CELLINK BIO X system, which enables automated droplet deposition with precisely controllable parameters such as extrusion pressure, dispensing time, and printing temperature, allowing for reproducible fabrication of 3D tumour–stroma microenvironments with accurately defined spatial positioning within well plates.

## Materials and methods

### Cell culture

OVCAR-4 ovarian cancer cells were transduced with lentivirus to stably express mApple RFP and nano-uciferase were maintained in RPMI supplemented with 10% FBS, 1% penicillin–streptomycin, L-glutamine, and 400 µg/mL G418 for selection. Primary ovarian cancer-associated fibroblasts (CAFs; Vitro Biopharma) were transduced with lentivirus to express telomerase and EGFP were cultured in DMEM containing 10% FBS, 1% penicillin–streptomycin, L-glutamine, and 0.5 µg/mL puromycin to maintain cell-selection.

### Preparation of Telecol and cell laden samples

Telecol-10 collagen (Advanced Biomatrix) was prepared according to the manufacturer’s instructions. Briefly, collagen, neutralising buffer, and sterile water were combined on ice to achieve physiological pH and the desired final concentration (6 mg/mL). For cell-laden bioinks, cell suspensions containing the required number of cancer cells, CAFs, or mixed ratios were transferred to sterile 1.5 mL tubes. Pre-cooled Telecol was then added to resuspend the cells, and pipetted up and down several times to form a homogenous cell-laden collagen bioink and remixed prior to printing.

### Bioprinting

Bioprinting was performed using the CELLINK BIO X in droplet-printing mode. The printer was homed and sterilised using industrial methylated spirit (IMS), followed by chamber UV sterilisation. To minimise collagen pre-gelation and droplet evaporation, prior to printing the printhead was cooled in a refrigerator to 4 °C. To further control the temperature, ice packs pre-cooled to 4 °C were also placed in the printing chamber. These were regularly replaced to maintain a cold environment for longer printing runs, the printhead temperature was set to 4 °C and the print-bed set to 0 °C. Extrusion pressure and extrusion-time were optimised to produce the smallest stable droplet volume. Bioprinted spheroids were imaged with a brightfield microscope (Olympus, CKX53). ImageJ was used to analyse the diameter of the spheroids. Once optimised, these parameters were applied for the full-plate print. The centre of each well in the 96-well plate was calibrated, followed by z-axis calibration, before initiating bioprinting. After printing, plates were immediately transferred to a 37 °C incubator to initiate collagen gelation. Constructs were then cultured at 37 °C and 21% O₂, and the outer wells were filled with PBS to minimise evaporation.

### Confocal microscopy

Confocal Microscopy was carried out on an Olympus FV3000. Sequential scanning mode was set to “Line” to minimize crosstalk between channels when imaging multiple fluorescent channels, ensuring that only one channel was excited and detected at a time. The confocal aperture was set to “Auto” and the laser power and detector voltage were optimized using the Hi/Lo intensity mode to prevent oversaturation of the images. Z-stacks were acquired by specifying the relative positions of the sample’s bottom and top surfaces, enabling the system to calculate the optimal number of slices required for the z-stack.

### Luciferase assay

Nano-Glo^®^ Luciferase Assay was performed following the manufacturer’s protocol to estimate relative cell number. Six samples were tested per plate for each assay condition evaluated. Briefly, the assay substrate was prepared by diluting one volume of Nano-Glo^®^ Luciferase Assay Substrate with 50 volumes of Nano-Glo^®^ Luciferase Assay Buffer. 100 µL of working solution was added to each well, and samples were mixed using a multichannel pipette to ensure uniform lysis. After a brief plate centrifugation, supernatant was transferred to an opaque 96-well plate and luminescence was recorded using a plate reader in luminescence mode.

### Drug treatment

In preliminary experiments using 2D cultured OVCAR-4 cells, the IC_50_ for carboplatin was determined to be 15 µM and this concentration used to guide the design of 3D experiments. Bioprinted plates containing (i) OVCAR-only, (ii) OVCAR and CAF at a ratio1:1, and (iii) OVCAR and CAF at a ratio of 1:3 constructs were treated with either vehicle control carboplatin. After 72 h, luminescence was measured using the Nano-Glo Luciferase assay as described above.

### Statistical analysis

Statistical analyses were performed using GraphPad Prism 9.5. For comparisons between multiple groups, one-way ANOVA was used when assessing the effect of CAF ratio or drug concentration as a single independent factor. All experiments were performed with a minimum of three biological replicates. Tukey’s multiple comparison test was applied following ANOVA where appropriate.

## Results and discussion

### Reproducibility of spheroid model with bioprinting

**Fig. 1:**
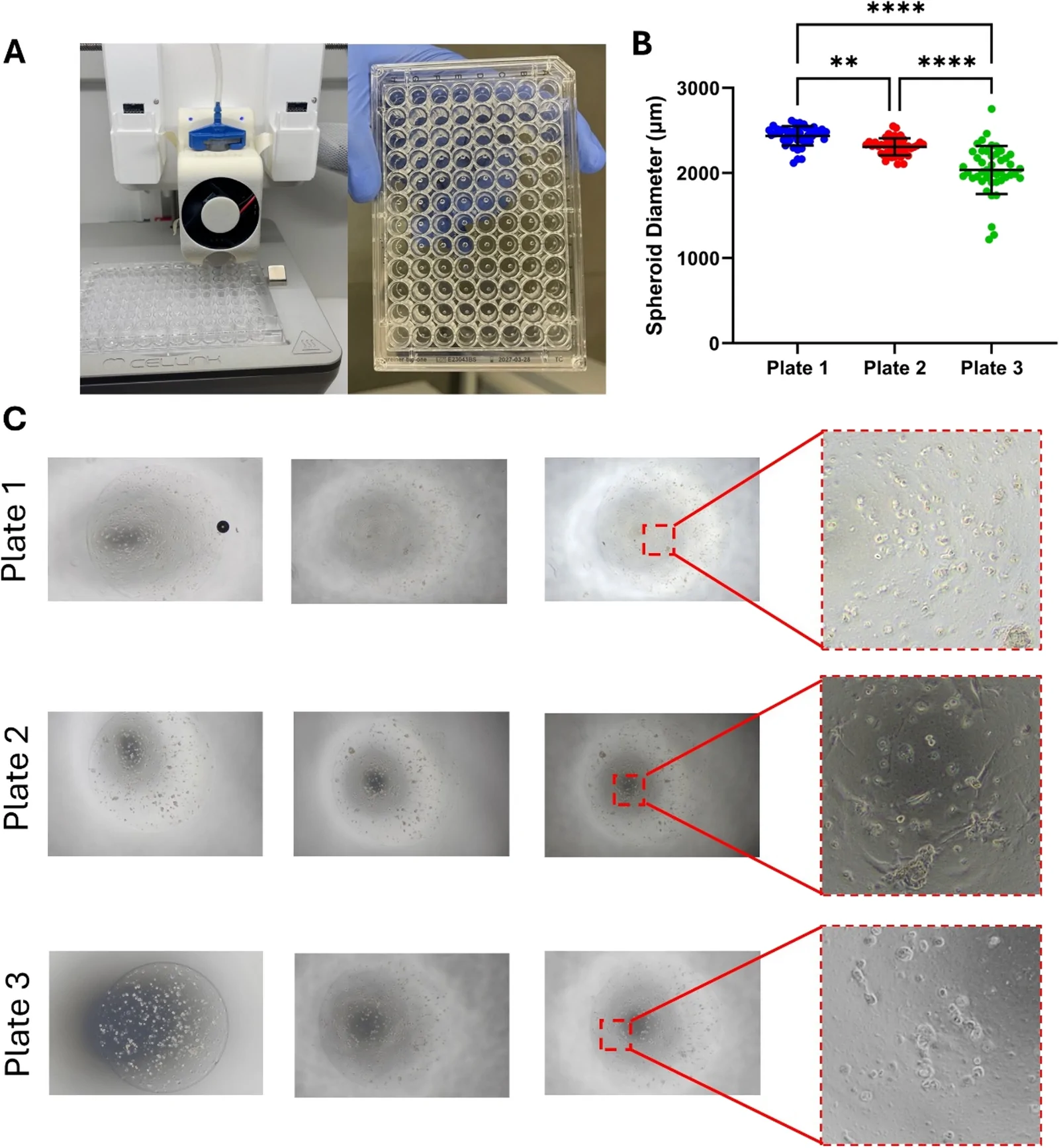
Characterising reproducibility of bioprinted spheroid models. Comparison of separate plates of spheroids prepared with different batches of cells on different days. (**A**) The system used to generate spheroids via droplet printing. (**B**) Plate-to-plate variability of spheroid diameter. Spheroid diameters on plates 1, 2 and 3 showed CVs of 4.6%, 4.3% and 19% respectively. Data is represented as mean ± SD with statistical significance determined via one-way ANOVA with Tukey’s multiple comparison test. **, *P* < 0.005; ****, *P* < 0.0001. (**C)** Representative brightfield images of bio-printed spheroids

We first assessed the uniformity of collagen-based spheroid droplets containing OVCAR-4 cells expressing nanoluciferase and bioprinted on a CELLINK BIO X. The diameter of the droplets was reasonably consistent within plates, although we did notice some inter-plate variability despite using the same method in each experiment (Fig. 1A). This likely reflects batch-to-batch variations in the viscosity of the bioink even if identical extrusion pressure and extrusion time are used. This could come about from minor differences in temperature in the printing chamber or different volumes of residual cell culture media in the cell pellets used to create the spheroids or incomplete mixing of the cells in the collagen which has to be performed rapidly to avoid the collagen solidifying. Consistent spheroid size ensures comparable diffusion gradients, mechanical properties, and cell numbers across replicates factors that directly influence proliferation, metabolism, and drug response [6, 7]. This is especially critical for drug testing, as spheroid size strongly affects drug penetration and cytotoxicity; variations in construct diameter can obscure treatment effects or lead to misleading interpretations. Although it may be useful to improve this in subsequent studies, we judged the method sufficiently robust to optimize the assay further by measuring nanoluciferase to estimate cell growth.

### Verification of the luciferase reporter and optimising the cell density within samples

**Fig. 2:**
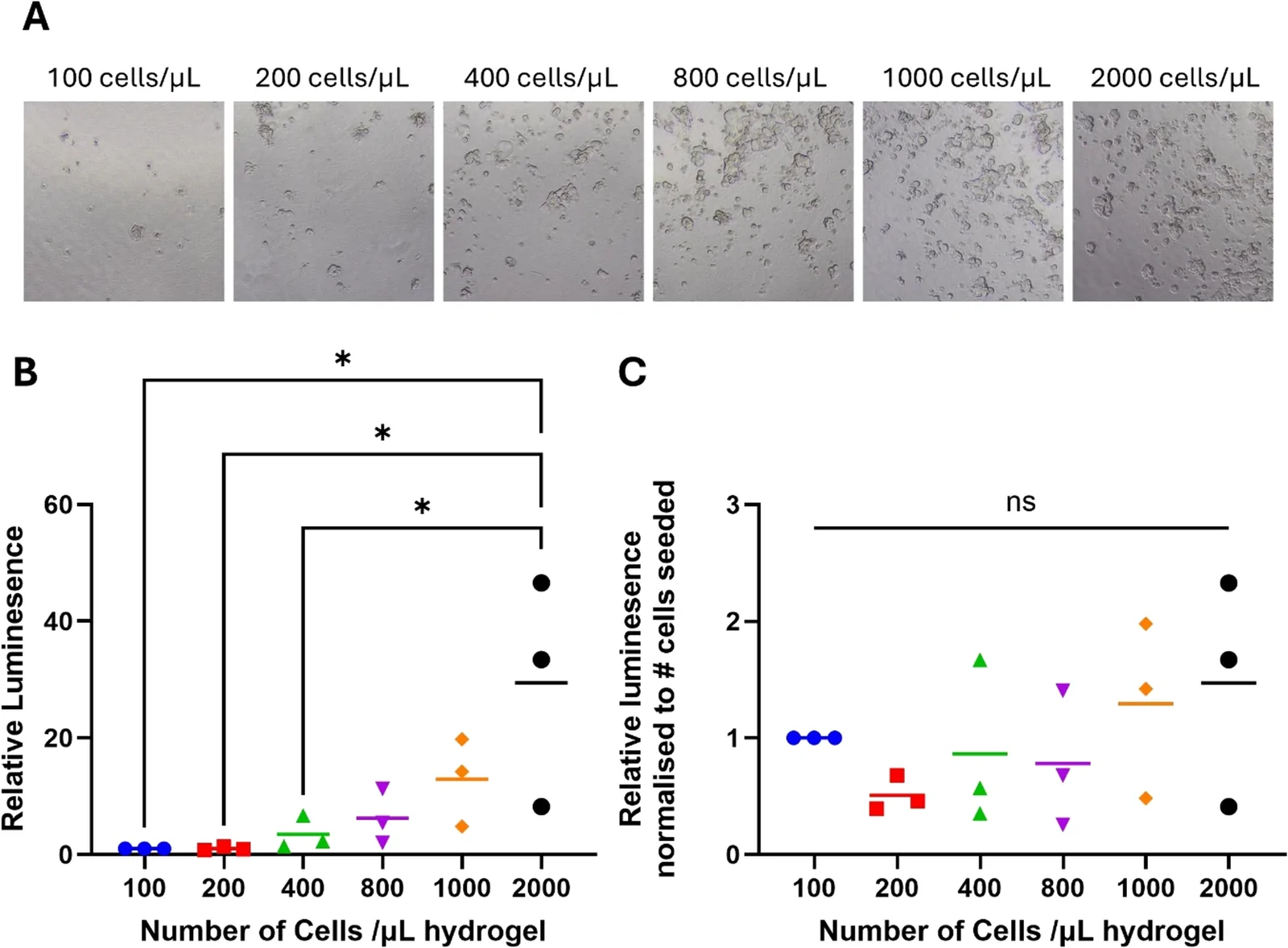
Optimisation of cell seeding density and verification of luciferase assay. (**A**) Representative brightfield images of increasing cell density within spheroids from one plate. (**B**) Graph of raw luminescence values vs. number of cells seeded, measured 3 days after cell seeding. (*n* = 3 independent experiments *, *P* < 0.05. (**C**) Graph of luminescence (normalised to number of cancer cells seeded) vs. number of cells seeded

To ensure that the bioprinted ovarian cancer model responded proportionally to changes in cancer cell density, we performed a cell-density optimization experiment using 100, 200, 400, 800, 1000, and 2000 OVCAR-4 cells per µL of collagen (Fig. 2A). A luciferase assay was performed 3 days after cell seeding. Increasing the number of cancer cells seeded resulted in a progressive increase in luminescence. (Fig. 2B). When 1000 cells were seeded per spheroid, the signal was 100-fold greater than that measured in spheroids without any cells. If the cells grew at the same rate, independent of the number of cells seeded, the increase in luminescence would be expected to be proportional to the number of cells seeded. To assess this, we normalised the luminescence signal to the number of cancer cells seeded. Although there was some variability, the luminescence per cell seeded did not significantly vary with initial seeding density. (Fig. 2C). However, we cannot rule out that had at higher cell seeding densities, growth was increased and our experiments were insufficiently powered to detect this. Possible explanations include denser constructs may have undergone proportionally greater proliferation due to cell-cell signalling or matrix compaction, or cell–cell signalling.

### Effect of CAFs on cancer cell growth

**Fig. 3:**
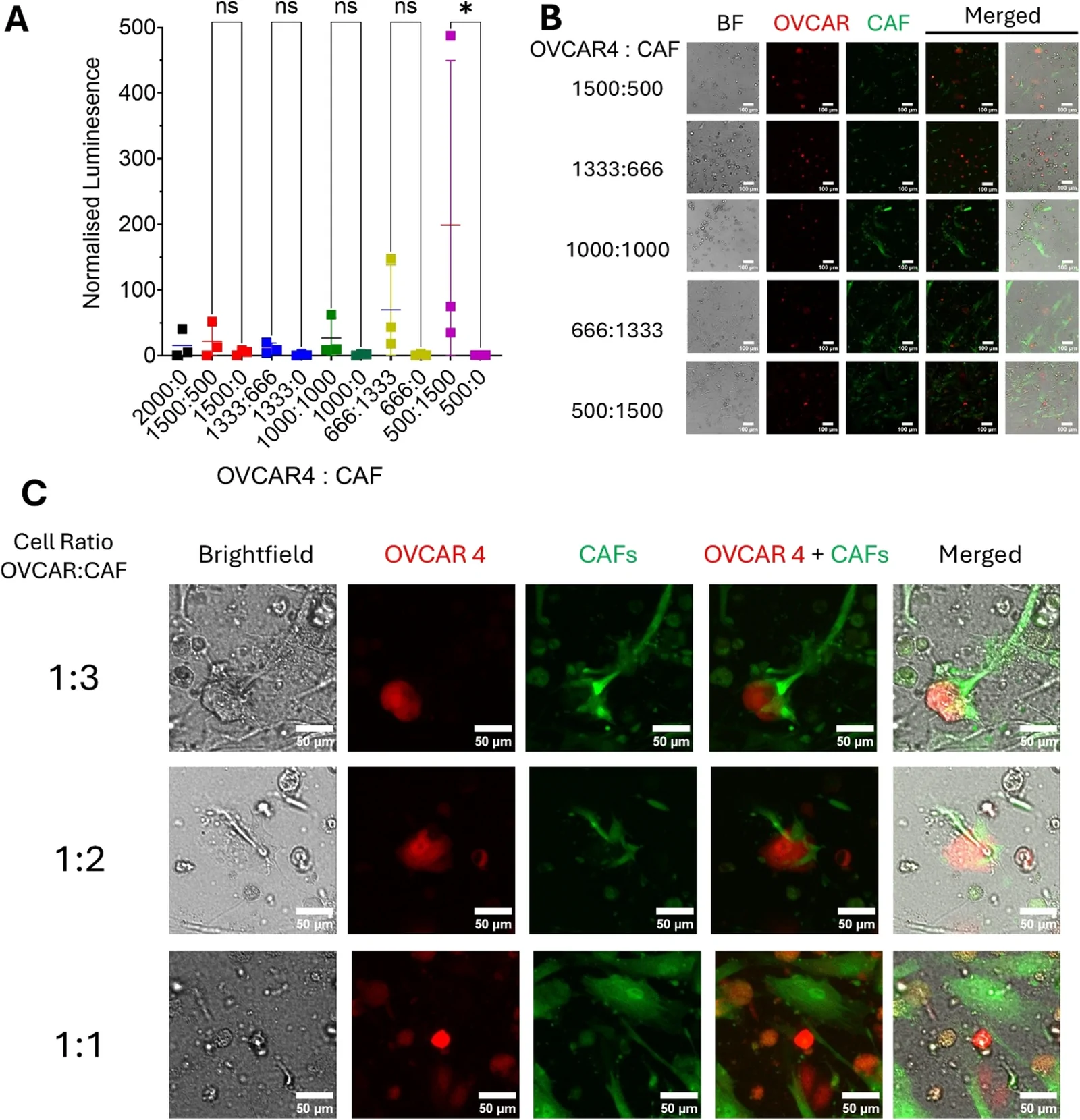
Mixed OVCAR-4 CAF collagen spheroids. (**A**) The effect of including CAF on the growth of OVCAR-4 cells in collagen spheroids was assessed by combining the indicated number of OVCAR4 and CAF cells when bioprinting the spheroid. After 72 h, luminescence was measured. The values from each experiment were normalised to the number of seeded cancer cells and to allow comparison between separate experiments. The data were expressed as a proportion of the signal measured in the sample containing 500 cancer cells without CAFs. Data is represented as mean ± SD with statistical significance determined via two-way ANOVA with Tukey’s multiple comparison test; ∗, *p* < 0.05). (**B**) OVCAR-4 (red) and CAF (green) cells were assessed by microscopy. (**C**) Higher magnification images show interactions between CAF (green) and OVCAR-4 cells (red)

To investigate how cancer-associated fibroblasts (CAFs) influence ovarian cancer growth in a 3D collagen microenvironment, we generated a series of bioprinted constructs in which OVCAR-4 and CAF cell number were systematically varied across a physiologically relevant range of CAF: cancer ratios while keeping the total number of cells per spheroid constant. Despite notable differences in absolute luminescence magnitudes across experiments, each replicate displayed an identical biological trend: increasing the proportion of CAF cells in the spheroids consistently enhanced cancer cell growth (Fig. 3A) and this was significant at the highest CAF: OVCAR4 ratio. CAFs are larger and more contractile than cancer cells, meaning that even equal numerical ratios can produce disproportionate increases in total physical cell mass and cell–cell interface area. This additional surface for direct contact may amplify growth-promoting cues. Confocal imaging further supported the presence of structurally organised OVCAR-4–CAF interactions within the constructs **(**representative images are shown in Fig. 3B-C). At higher CAF ratios, CAFs frequently arranged themselves into capsule-like structures around cancer cells, rather than remaining evenly dispersed. This CAF encapsulation suggests the formation of a stromal niche in which fibroblasts physically enclose tumour aggregates while simultaneously compacting the collagen matrix, a phenomenon well documented in CAF–cancer interactions, where activated fibroblasts modulate mechanical signalling and reorganise tumour morphology as shown by Barbazan et al. [8]. This architecture likely stabilises cancer–CAF interactions, concentrates growth-promoting signals, and creates microenvironments of altered mechanical tension and soluble factor gradients. Thus, the increased growth of the OVCAR-4 cells may have resulted from direct cell-cell contact. Potential mechanisms for this include activation of pathways involving integrins, cadherins, and juxtacrine signalling networks such as Notch or Eph–ephrin [9–11]. It is also possible that secreted factors also contributed to this enhancement. CAFs secrete a wide range of paracrine mediators (IL-6, HGF, TGF-β, CXCL chemokines) known to stimulate tumour cell proliferation. CAFs also remodel the surrounding collagen through MMP secretion and mechanical remodelling that requires actomyosin contractility induced by α5 and α3 integrin/Rho-Rock signalling [5, 12, 13]. Such changes can enhance cancer growth by increasing mechanotransducive signalling (e.g., YAP/TAZ activation), improving nutrient distribution, or generating compressed niches that promote tumour clustering [14]. In pancreatic cancer, metabolites released by CAF can also support the growth of cancer cells [15].

### Response to carboplatin

**Fig. 4:**
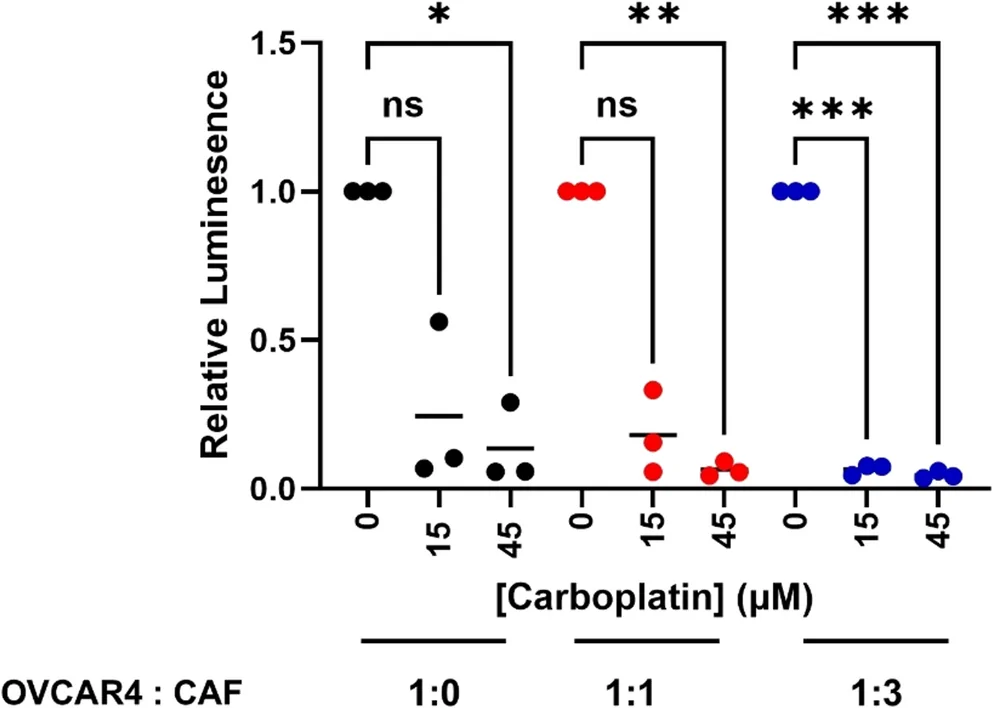
Bioprinted model response to carboplatin treatment at a concentration equal to its IC_50_ or 3 x IC_50_ (15 and 45 µM, respectively) measured using cell monolayers. Bioprinted collagen spheroids containing either cancer cells alone or combined with CAF at the indicated ratio were exposed to carboplatin for 72 h and the relative number of surviving cancer cells determined by measuring NanoLuc. Luminescence values were normalised to seeded cancer cell number and then expressed relative to the untreated cancer-only control within each experiment setting to allow comparison of the effect of carboplatin at different OVCAR-4: CAF ratios. *, *P* < 0.05; **, *P* < 0.005; ***, *P* < 0.001

To evaluate whether CAFs modulate the chemotherapeutic response of ovarian cancer cells in the bioprinted Telecol collagen system, OVCAR-only, OVCAR: CAF 1:1, and OVCAR: CAF 1:3 co-cultures were treated with carboplatin at a concentration equal to its IC_50_ (15 µM) and 3× IC_50_ (45 µM) measured in preliminary experiments with cells grown as monolayers. Across all three independent experiments, carboplatin treatment produced a strong and significant cytotoxic response, reflected by substantial decreases in luminescence in both cancer-only and CAF-containing conditions (Fig. 4). This demonstrates that the effect of the drug can be measured in the cancer cells specifically in a spheroid containing a mixed population of cells because the nano-luciferase is only expressed in the cancer cells. The CAFs again increased the growth of the ovarian cancer cells but we did not observe any chemoprotection under any condition tested. If anything, the inclusion of the CAFs increased the response to carboplatin, but this did not reach statistical significance. The absence of CAF-mediated chemoresistance in our model contrasts with several reports where CAFs actively enhance drug resistance. For example, CAFs in gastric cancer promote 5-FU resistance through IGF1/IGF1R–PI3K–AKT signalling, reducing apoptosis and increasing clonogenic survival in tumour cells [16]. Likewise, CAF-driven chemoprotection has been attributed to SDF-1/CXCL12–YAP/TAZ activation and the release of pro-survival exosomes in multiple other tumour contexts [17]. In contrast to our results, several reports have claimed that CAFs decrease the sensitivity of ovarian cancer cells to platinum-based drugs specifically in ovarian cancer cells [18–21]. There are several possible explanations for this difference.

Firstly, several of these reports have added CAF-conditioned medium to ovarian cancer cells in 2D and shown that this decreases the response to carboplatin or cisplatin. In contrast, our approach of labelling the OVCAR-4 cells with nano-luciferase allows true 3D co-cultures to be evaluated where direct cell-cell contact can take place, more closely recapitulating an authentic tumour. The confocal imaging data showed that CAFs formed encapsulating structures around cancer clusters, which may generate a stromal niche that enhances growth, but which is insufficient to form a diffusion barrier sufficient to impair drug penetration. In collagen-rich matrices such as Telecol, CAF-driven matrix contraction can increase porosity or create aligned fibre channels, which may facilitate, rather than impede, drug diffusion through the spheroid. Additionally, CAF-induced compaction may increase local cell density and thus increase the number of cells directly exposed to cytotoxic stress, contributing to the comparable or heightened carboplatin sensitivity observed. In the collagen-based bioprinted system developed in this work, however, CAFs may remodel the matrix through MMP activity and contractility, creating a compact but non-occlusive microenvironment that does not activate strong pro-survival signalling pathways or impede drug penetration potentially explaining why CAFs enhanced growth yet failed to confer resistance to carboplatin. Further studies will be necessary to address these issues, but we consider that the co-culture method we have developed provides a better model to achieve this than using CAF conditioned media.

Secondly, chemotherapeutic drugs are more effective on proliferating cells, so the increased proliferation rate caused by the fibroblasts might be expected to increase sensitivity to carboplatin, balancing any chemoprotection. Thirdly, some ovarian cancer cells may not respond to CAF-mediated chemoprotection. Fourthly, CAFs themselves are likely to be sensitive to carboplatin, perhaps more so than cancer cells, and their partial depletion could remove supportive signalling inputs, indirectly sensitising adjacent cancer cells. Finally, we acknowledge that our failure to observe CAF chemoprotection may reflect our choice of carboplatin concentrations. Ideally a full range of carboplatin concentrations would be assessed and over a protracted period to allow the emergence of resistance which we may have failed to detect in our 72-hour assay. Overall, these findings highlight that CAF-driven growth support can be observed in the absence of CAF-driven chemoprotection and that cell-cell contact and the ECM context may have a major influence on whether CAFs promote drug resistance.

Our study has a number of deficiencies. Firstly, we did not test the impact of bioprinting on the viability of the cells (although there was no morphological evidence of an issue). Secondly, we used nanoluciferase as a surrogate marker of cell number, and this may not give a true representation of viable cell number because differences in luminescence could also reflect altered expression of the reporter. Thirdly, we have not assessed whether the CAF cause matrix remodelling and we have not measured the effect of the cancer cells on the growth of CAFs. Forthly, we have not evaluated whether chemoresistance induced by CAFs emerges over longer culture periods. Finally, we have not evaluated the impact of carboplatin on the CAFs because the nanoluciferase reporter specifically measures the cancer cells. These issues will be addressed in subsequent experiments.

## Conclusion

In this study, we developed and validated a droplet-based bioprinted ovarian cancer model incorporating NanoLuc-expressing OVCAR-4 cells and primary cancer-associated fibroblasts embedded within Telecol collagen. This system has allowed direct measurement of OVCAR-4 cell growth in a mixed culture system. CAF enhanced cancer cell growth, and the CAFs frequently organised into encapsulating or shell-like structures around cancer aggregates. However, further optimization of the method is desirable to reduce the variability we observed and re-evaluate whether CAF-mediated chemoprotection can be observed in our model.

## Data Availability

All processed data in included in the manuscript. Original data are available on request.
